# Promoting a Sleep-friendly Environment by Minimizing Overnight Room Entries

**DOI:** 10.1097/pq9.0000000000000668

**Published:** 2023-12-12

**Authors:** Lauren M. McDaniel, Nilesh Seshadri, Elizabeth A. Harkins, Megan Keydash, Alice Pan, Laura M. Sterni, Shawn L. Ralston

**Affiliations:** From the * Department of Pediatrics, Seattle Children’s Hospital, Seattle, Wash.; † Department of Pediatrics, Children’s Hospital of Philadelphia; ‡ Department of Pediatrics, Johns Hopkins Children’s Center; § Department of Pediatrics, University of North Carolina Children’s Hospital

## Abstract

**Introduction:**

Despite its importance in illness recovery, the sleep of hospitalized children is frequently interrupted. This quality improvement intervention aimed to reduce overnight room entries by minimizing unnecessary interventions.

**Methods:**

This study occurred at a university-affiliated children’s hospital on the hospital medicine services from March 26, 2021, to April 14, 2022. The intervention included order set changes and the implementation of a rounding checklist designed to address factors most closely associated with sleep disruption and overnight room entries. The outcome measure was overnight (10 pm to 6 am) room entries, counted using room entry sensors. Process measures reflected the intervention targets (overnight vital sign orders, medication administration, and intravenous fluid use). The method of analysis was statistical process control charting.

**Results:**

After identifying special cause variation, the average number of overnight room entries decreased from 8.1 to 6.8, a 16% decrease. This decrease corresponded with the implementation of a rounding checklist. However, there continued to be variability in average room entries, suggesting a process lacking ongoing stability. During this period, avoidance of overnight medications and intravenous fluid increased by 28% and 17%, respectively.

**Conclusions:**

Implementing a rounding checklist to a broad patient population decreased overnight room entries. However, future work is needed to better understand the factors associated with sustaining such an improvement.

## INTRODUCTION

Adequate sleep is essential to recovery from illness.^1,2^ Yet, sleep is interrupted frequently in the hospital setting.^3,4^ Others have found that the most common reason for awakening after sleep initiation is a provider entering the room^5^ and that the rooms of hospitalized pediatric patients are entered an average of 10 times per night.^4^ Caregivers commonly identify vital signs and overnight medication administration as disruptive to sleep.^6,7^ These same interruptions are also associated with increased room entries.^8^ The practice of routinely obtaining vital signs overnight on all patients is not evidence-based,^9^ and many commonly prescribed medications can be safely retimed to avoid nighttime administration.^10^

This quality improvement (QI) project aimed to improve the sleep of patients admitted to pediatric hospital medicine services by reducing overnight (10 pm–6 am) room entries by 30% within 1 year (by April 15, 2022). We sought to achieve this by minimizing unnecessary overnight interventions [ie, vital sign measurements, medication administration, and intravenous fluid (IVF) use].

## METHODS

### Context

Johns Hopkins Children’s Center is a 205-bed tertiary pediatric care academic center in Baltimore, Maryland. All rooms are private, with 1 patient per room. All patients on hospital medicine services are cared for by resident physicians supervised by attending hospitalists. There are no in-house attending hospitalists at night. Hospital medicine teams typically consist of 2 senior residents, 2 interns, and 2–3 medical students. A charge/lead nurse rounds with the inpatient teams and represents the questions and concerns of bedside nurses. A pharmacist intermittently rounds with the teams when available. Family-centered, bedside rounding occurs for most patients.

### Interventions

**Fig. 1.**
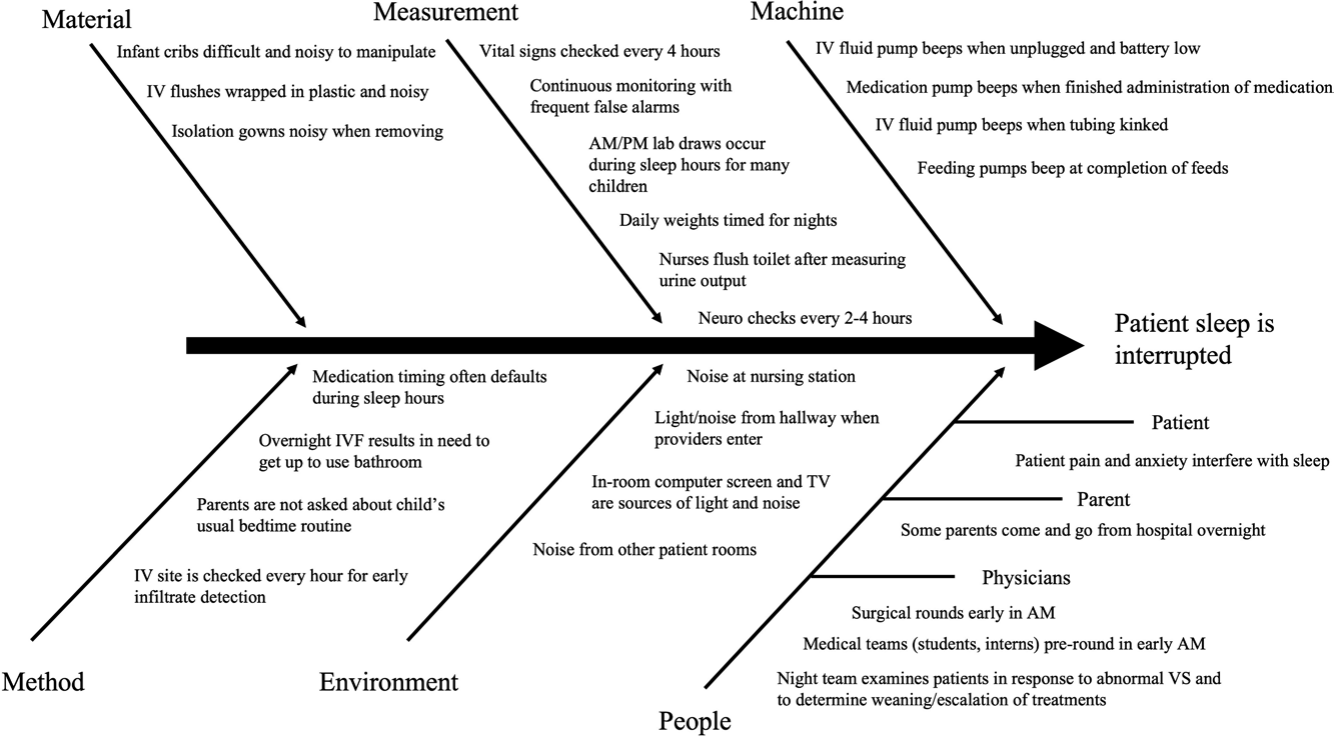
Fishbone diagram. IV indicates intravenous; neuro, neurologic; VS, vital signs.

A hospitalist, pulmonologist, resident physician, 2 inpatient nurses, and a pharmacist formed the interprofessional QI leadership team. At the start of the project, this team recruited additional physicians, nurses, a social worker, a patient experience coach, a patient and family advocate, and a parent representative to join multidisciplinary discussions of barriers to adequate sleep in the hospital setting. From these discussions, the team generated a fishbone diagram of contributing factors to interrupted patient sleep (Fig. 1).

### Provider Education

Physician and nursing project leads conducted education on the importance of sleep in the hospital setting. Physician education occurred at a resident noon conference and a department faculty meeting. Nursing education occurred in each of the acute care units. During these educational sessions, we highlighted the ability to modify vital signs orders to “every 4 hours during waking hours (0600–2200)” within the electronic medical record (EMR).

### EMR Modification [Plan-Do-Study-Act (PDSA) Cycle 1—April 27, 2021]

Before this project, the default (and only) vital sign order in the general pediatrics admission order set was “every four hours (Q4H)” without the option to order a different frequency. Ordering providers had to modify the order manually to change the frequency from the default.

We added the option for vital signs “every 4 hours during waking hours (0600–2200)” and “every 8 hours” to the general pediatrics admission order set. We additionally attempted to add a forced stop by removing the default selection of vital signs Q4H, but we could not achieve consensus among institutional order set stakeholders. Those hesitant to make this change expressed concerns about whether trainees could appropriately distinguish which patients were at the highest risk of decompensation and required more frequent monitoring at admission.

### Rounding Checklist (PDSA Cycle 2–4—January 11, 2022, February 8, 2022, and March 8, 2022)

**Fig. 2.**
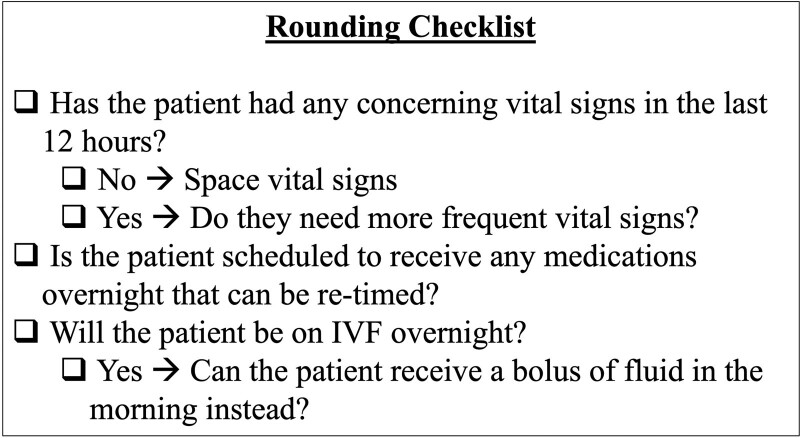
Rounding checklist.

The next stages of the QI intervention occurred on the hospital medicine services and involved the development of a rounding checklist. We added a question to each PDSA cycle’s rounding checklist (each month) until there were 3 total questions (Fig. 2). The rounding checklist focused on minimizing the most disruptive overnight interventions. Previously, caregivers subjectively reported vital signs, medication administration, and alarms (eg, from IVF pumps) as most disruptive to sleep.^6,7^ Objectively, we studied overnight room entries at our institution. We found that both vital signs ordered Q4H and overnight medication administration were each independently associated with a 1.3-fold increase in room entries per night.^8^ We utilized these subjective and objective findings to generate the checklist.

The resident QI lead on the project educated residents on the checklist with reminders at the start of each clinical block. Residents received copies of the checklist electronically and in paper form. Due to the time required to make EMR modifications, we could not incorporate the checklist into the medical record before initiating these PDSA cycles. The senior resident leading rounds were responsible for reviewing the checklist with the team. The rounding team encouraged input from the patient, caregiver, nurse, and pharmacist on decisions related to order changes.

### Measures

#### Outcome Measure

The primary outcome measure was room entries between 10 pm and 6 am. We tracked this metric using wireless entry sensors (YoLink, Irvine, Calif.). We placed sensors on 18 of the 80 doors on the medical-surgical units. We selected doors for sensor placement based on the strength of the wireless signal. Via hub devices, the sensors were communicated to a smartphone application where we could access the dates and times each door opened and closed.

We defined a room entry as sensor activation for entry followed by sensor activation for closure. There were 2 exceptions to this: (1) if a door opening and closure occurred within 30 minutes of another door opening and closure, and each opening and closure occurred within fewer than 30 seconds, this was counted as a single room entry (as opposed to 2 entries) to account for the likelihood of the door being closed while the provider remained in the room. (2) If a door was left open (no door closure after a door opening) for more than 2 hours, we excluded the night to prevent systematic underestimation of room entries, as multiple entries/exits were likely to have occurred while the door was left open. We similarly excluded sensors that lost wireless signals.

We accessed and logged sensor data Monday through Friday from March 26, 2021, to April 14, 2022. To ensure patients admitted to rooms with room entry sensors met inclusion criteria, we conducted chart reviews daily in real time. Weekends were excluded due to staffing limitations for manual chart review. Because the QI interventions specifically targeted the hospital medicine teams, we only accessed room entry data on patients admitted to a hospital medicine service. All patients (of all ages) admitted to hospital medicine services into rooms with room entry sensors were included. To minimize the influence of outlier nights on the outcome measure, we made the a priori decision to exclude the first night of admission/transfer to the floor and any nights involving rapid response team activation or codes.

Front-line providers were aware of the QI intervention; however, they were neither specifically educated nor blinded to the presence or purpose of the room entry sensors.

#### Process Measures

We reviewed patients’ medical records meeting inclusion criteria and tracked the ordered frequency of vital signs (Q4H versus less frequently) as a process measure. No patients had vitals measured more frequently than every 4 hours. We also tracked whether patients received medications and IVF between 10 pm and 6 am. These metrics were recorded as yes/no and confirmed based on medication administration record data.

#### Balancing Measures

We reviewed all rapid response/code events and patient safety reports from the acute medical-surgical units during this study period to assess for unintended consequences of our intervention. Although nights involving rapid responses/ICU transfers were excluded from the outcome measure, we still reviewed these events for the balancing measures.

### Data Analysis

We used u (outcome measure) and p (process measures) statistical process control charts with standard upper and lower control limits (±3 SD). We applied Western Electric rules^11^ to identify special cause variation. The centerline was shifted for the outcome (room entries), and the process measures (overnight medications and IVF) after 8 consecutive points fell above or below the centerline.

### Ethical Considerations

The Johns Hopkins University institutional review board approved this quality improvement project as nonhuman subjects research (IRB00278171).

## RESULTS

Out of a total of 4950 eligible patient nights, 61% were excluded because the patient was not admitted to a hospital medicine service, 22% because the night was the first night of admission/transfer, 16% because the door was open for more than 2 hours or the sensor lost wireless signal, and <1% because of a rapid response or code.

Baseline data collection began on March 23, 2021. There were 17 unique patients and 29 patient nights represented in these data. The intervention period began on April 27, 2021, and included 93 unique patients and 203 patient nights. Unfortunately, due to technical difficulties with the room entry sensors, there was a 2-month gap in data collection between August 10, 2021, and November 10, 2021.

### Outcome Measure—Room Entries

**Fig. 3.**
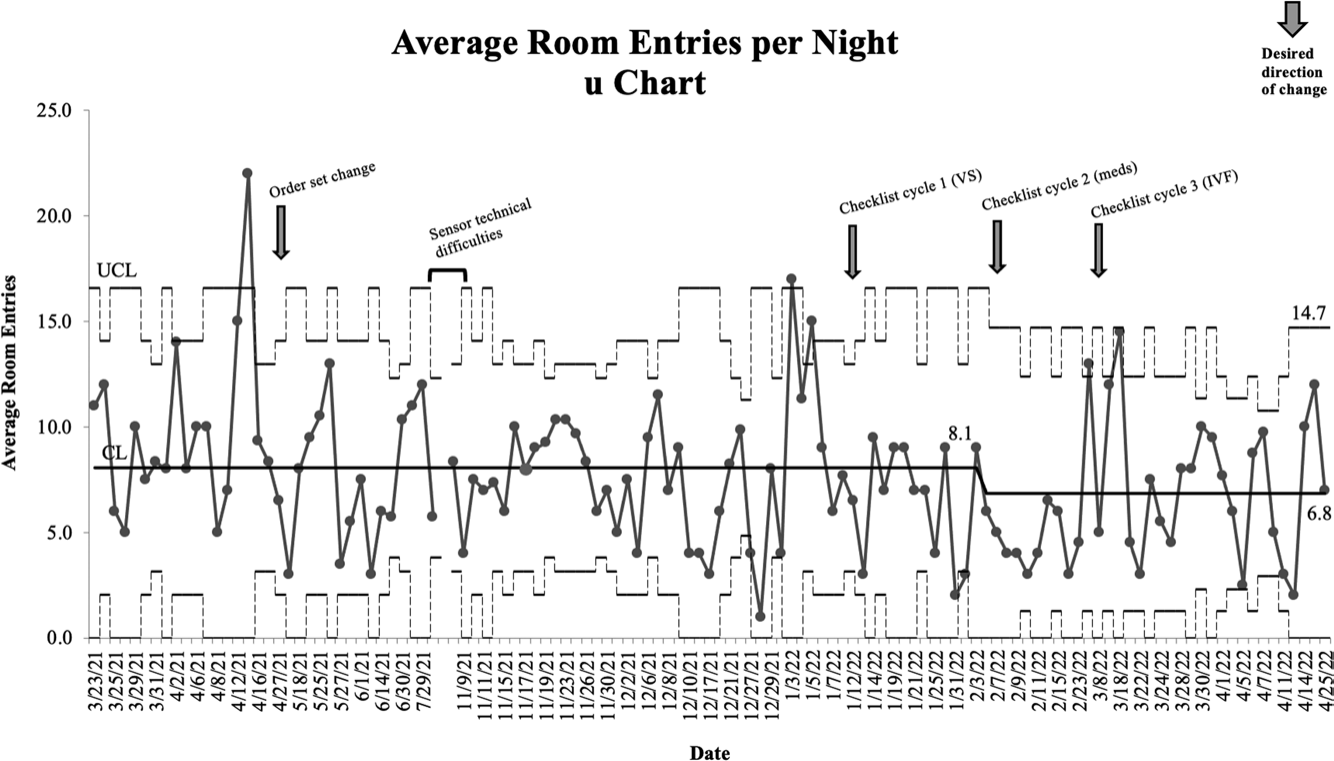
Average room entries per night (u chart). meds, medications; VS, vital signs.

Before the identification of special cause variation, there was an average of 8.1 room entries per night. Special cause variation occurred on February 3, 2022; the centerline shifted to an average of 6.8 room entries per night (Fig. 3). This change represented a 16% decrease in room entries. This period overlapped with the implementation of the rounding checklist. The first checklist PDSA cycle began on January 11, 2022, and the third began on March 8, 2022. Three weeks after the start of the third checklist PDSA cycle (March 18, 2022), the average room entries increased to the upper control limit with an average of 14.7 room entries before returning to the baseline by March 21, 2022. The order set change earlier in the QI intervention was not associated with an effect on room entries.

### Process Measures—Ordered VS Frequency, Administration of Scheduled Medications Overnight, and Use of IVF Overnight

**Fig. 4.**
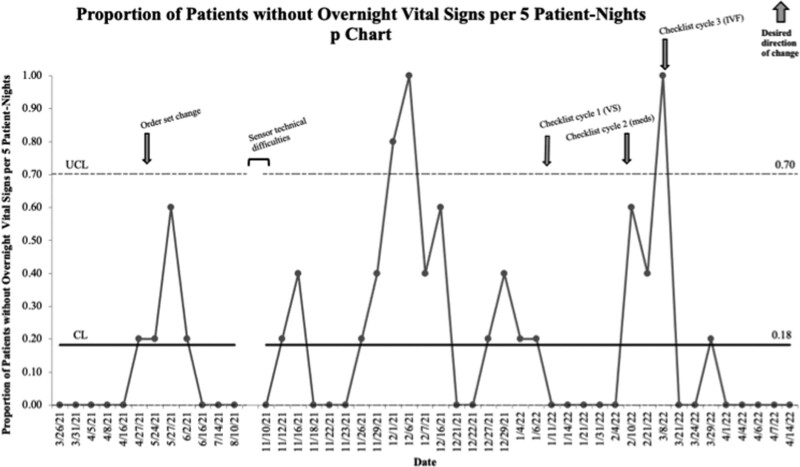
Proportion of patients without overnight vital signs per 5 patient nights (p chart). meds, medications; VS, vital signs.

The average proportion of patients without overnight vital signs per 5 patient nights was 18% throughout the study (Fig. 4). The first cycle of the checklist (specifically addressing vital signs) began on January 11, 2022. Before and after checklist implementation, there were instances of special cause variation (as defined by 1 point above the UCL); however, we could not sustain change in each instance.

**Fig. 5.**
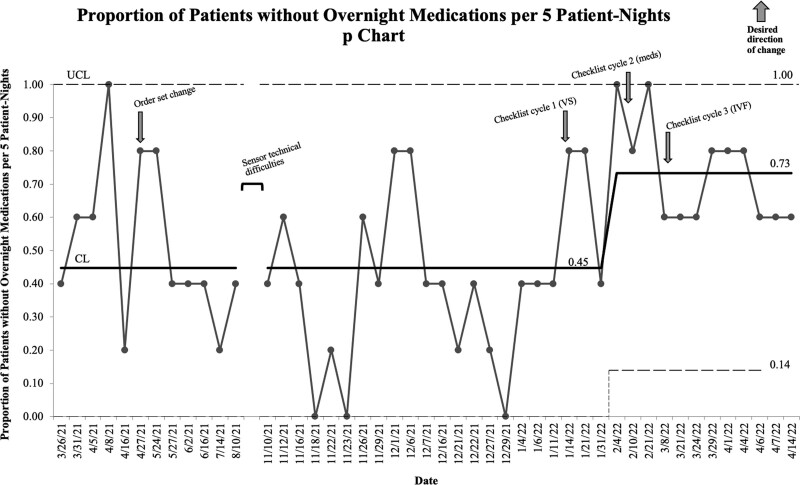
Proportion of patients without overnight medications per 5 patient nights (p chart).meds, medications; VS, vital signs.

The checklist cycle involving retiming of medications began on February 8, 2022. When analyzing the proportion of patients without overnight medications administered per 5 patient nights, special cause variation (as defined by 8 consecutive points above the control limit) occurred on February 4, 2022 (Fig. 5). This change represented a 28% increase in patients not receiving medications overnight. We sustained this change throughout the remainder of the study.

**Fig. 6.**
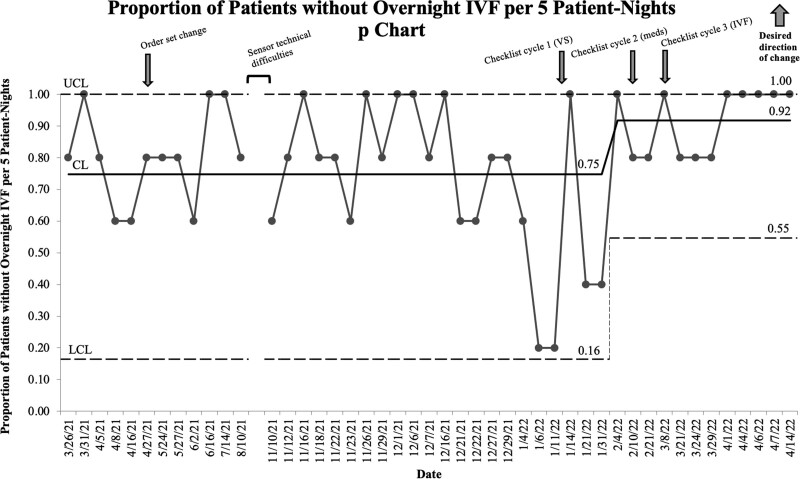
Proportion of patients without overnight IVF per 5 patient nights (p chart).meds, medications; VS, vital signs.

Special cause variation (8 consecutive points above the control limit) for the proportion of patients without overnight IVF administered per 5 patient nights occurred on February 4, 2022, representing a 17% increase in patients not receiving overnight IVF (Fig. 6). The third cycle of the rounding checklist addressing IVF use began on March 8, 2022. We sustained the change in overnight IVF administration throughout the study.

### Balancing Measures—Rapid Response/Code Events and Patient Safety Reports

There were no rapid response/code events or patient safety reports related to this intervention.

## DISCUSSION

This QI intervention achieved a 16% decrease in overnight room entries in hospitalized pediatric patients admitted to general medical services. The timing of this decrease tracked with the implementation of a rounding checklist, which we designed to address factors most closely associated with sleep disruption^6,7^ and overnight room entries in our hospital.^8^ Despite this decrease, there continued to be wide variation in average room entries, suggesting a process lacking ongoing stability. Unlike the rounding checklist, the admission order set modification did not affect the outcome measure. This finding may relate to our inability to remove the default selection of Q4H vital signs within the admission order set. The influence of a default option on provider behavior is well documented.^12^

We were unable to sustain a change in the vital sign process measure. It is possible that the clinical stability of the patients captured within the study necessitated more frequent vital sign monitoring. However, throughout the project, we encountered the most hesitation (predominantly from physicians) to changing the frequency of vital signs due to concerns about missing early signs of clinical deterioration. Our study attempted to overcome this barrier by suggesting that patients without concerning vital signs for the preceding 12 hours should be considered candidates for less frequent vital signs. Others have utilized pediatric early warning scores for this purpose.^13^ Future work will need to understand further and target provider concerns.

We identified and sustained special cause variation with the medication and IVF process measures, corresponding to the timing of special cause variation identified in our outcome measure, room entries. Additionally, there appeared to be a relationship between both process measures and the timing of implementation of the rounding checklist.

Others have conducted QI interventions intending to minimize unnecessary disruptions to sleep.^7,10,13–15^ Cooke et al,^7^ Lee et al,^13^ and Lin et al^14^ similarly targeted overnight vital sign orders; however, Cooke et al only targeted blood pressure, while Lee et al targeted blood pressure and temperature measurement (in addition to laboratory collection timing). Lin et al limited the study population to pediatric patients hospitalized with failure to thrive or hyperbilirubinemia. Mozer et al^10^ targeted medication administration times but limited the intervention to 4 oral antibiotics. Like our intervention, Ramazani et al^15^ also used a rounding checklist but targeted only laboratory collection timing. These studies tracked order changes or subjective measures of sleep disruption (caregiver report) as their outcome measures.

Using a primary outcome metric that is objective and a closer proxy for sleep interruption strengthened our study. Previous studies have been restrictive in their inclusion criteria and targets of intervention. We attempted to address this gap in the literature by capturing a broad patient population by not limiting our inclusion criteria by illness severity. In addition, while designing our rounding checklist intervention, we studied the objective factors that result in the largest increases in room entries at our institution^8^ to prioritize the interventions most likely to result in improvement.

A major challenge we faced with implementing our intervention was the lack of a preexisting rounding checklist. Thus, we overcame the barrier of incorporating a checklist into the rounding workflow. The time required to make EMR and policy changes at our institution also limited our team. Other institutions with preexisting structured checklists, particularly those that have checklists integrated into the EMR and those with processes and/or pathways allowing for more streamlined improvements in care, might be able to replicate our work and demonstrate more substantial and sustainable improvements.

### Limitations

Our study did not track checklist use as a process measure. Process measures more closely aligned with our outcome measure were less vulnerable to the Hawthorne effect. Tracking checklist use would have required observation of rounds. However, it is possible that the effects on our outcome and process measures were not directly related to using the checklist and instead reflected other trends or factors. In general, there was significant instability in our outcome measure, and due to the lack of sustained change in our process measures, establishing cause and effect is impossible. Sensors were visible to front-line providers and could theoretically influence provider behavior; however, the sensors were very small, not easily visualized, and most front-line providers were unaware of their purpose.

Additionally, the sensors did not allow us to distinguish between entries by hospital staff and entries by caregivers; however, the number of caregiver room entries theoretically should have been similar throughout the study and should not have been significantly influenced by our interventions. Although we did experience 2 months of technical difficulties with the sensors, we did not implement any additional interventions during this period. Therefore, it is unlikely that our results would have been different with adding these data. Finally, the fact that this was a single-center intervention with private patient rooms limits the generalizability of our work.

## CONCLUSIONS

Implementing a rounding checklist to a broad patient population decreased overnight room entries. However, future work is needed to better understand the factors associated with reliably sustaining such an improvement.

## DISCLOSURE

The authors have no financial interest to declare in relation to the content of this article.
